# The Effect of Manual Therapy Plus Exercise in Patients with Lateral Ankle Sprains: A Critically Appraised Topic with a Meta-Analysis

**DOI:** 10.3390/jcm11164925

**Published:** 2022-08-22

**Authors:** Rocco de Ruvo, Giuseppe Russo, Francesco Lena, Giuseppe Giovannico, Christoper Neville, Andrea Turolla, Monica Torre, Leonardo Pellicciari

**Affiliations:** 1 Fondazione Centri di Riabilitazione “Padre Pio Onlus”, 71013 San Giovanni Rotondo, Italy; 2 Department of Medicine and Health Science “Vincenzo Tiberio”, University of Molise, 86100 Campobasso, Italy; 3 IRCCS INM Neuromed, 86077 Isernia, Italy; 4 Department of PT Education, Upstate Medical University, Syracuse, NY 13210, USA; 5 Dipartimento di Scienze Biomediche e Neuromotorie—DIBINEM, Università degli Studi di Bologna, 40126 Bologna, Italy; 6 IRCCS Azienda Ospedaliero-Universitaria, 40138 Bologna, Italy; 7 Sanstefar Abruzzo Riabilitazione, 65100 Pescara, Italy; 8 IRCCS Istituto delle Scienze Neurologiche di Bologna, 40126 Bologna, Italy

**Keywords:** ankle injuries, exercise therapy, musculoskeletal manipulation, exercise

## Abstract

A high percentage of patients with lateral ankle sprains report poor outcomes and persistent neuromuscular impairment leading to chronic ankle instability and re-injury. Several interventions have been proposed and investigated, but the evidence on manual therapy combined with therapeutic exercise for pain reduction and functional improvement is still uncertain. The purpose was to study the effectiveness of adding manual therapy to therapeutic exercise in patients with lateral ankle sprains through a critically appraised topic. The literature search was performed in PubMed, PEDro, EMBASE and CINAHL databases, and only randomized clinical trials were included according to following criteria: (1) subjects with acute episodes of lateral ankle sprains, (2) administered manual therapy plus therapeutic exercise, (3) comparisons with therapeutic exercise alone and (4) reported outcomes for pain and function. Three randomized clinical trials (for a total of 180 patients) were included in the research. Meta-analyses revealed that manual therapy plus exercise was more effective than only exercises in improving dorsal (MD = 8.79, 95% CI: 6.81, 10.77) and plantar flexion (MD = 8.85, 95% CI 7.07, 10.63), lower limb function (MD = 1.20, 95% CI 0.63, 1.77) and pain (MD = −1.23; 95% IC −1.73, −0.72). Manual therapy can be used with therapeutic exercise to improve clinical outcome in patients with lateral ankle sprains.

## 1. Introduction

Lateral ankle sprains (LAS) are a common musculoskeletal injury occurring in the general population, especially during sport activities [1,2] such as rugby, tennis, football, volleyball and basketball [3]. The prevalence of LAS is estimated to be as high as 45% of athletes across their sport participation [4]. The etiopathogenetic mechanism is typically described as an acute trauma with the ankle positioned with an inversion, or with an inversion and plantar flexion [5,6,7]. About 32% to 74% of patients with a previous LAS present multiple mechanical and neuromuscular impairments, which may persist long after the healing of tissues and at the end of rehabilitation treatment [8,9,10,11]. Patients with an LAS have been identified to have a reduction in the thickness of the plantar fascia [12], and a reduced cross-sectional area of the peroneus brevis [13] when compared to healthy subjects. Furthermore, patients with an LAS present an alteration in activation patterns for select lower limb proximal muscles [14]. The high percentage of re-injury, ranging from 30% to 40% [15], may be due to inadequate rehabilitation [16] and an early return to playing a sport [17]. LAS injuries are associated with high social economic costs related to diagnosis, rehabilitation and reduced work productivity. In the United Kingdom, Cooke, Lamb, Marsh and Dale [18] reported an average of 6.9 days of lost paid work due to LAS. This equates to an additional £805 in lost productivity costs for each injury in addition to the £135 of direct healthcare costs. The combination of high incidence and high direct and indirect costs makes the economic burden of lateral ankle sprain injuries indisputable [19].

A widely agreed upon therapeutic protocol for LAS management remains controversial, as the current clinical guidelines suffer from methodological weaknesses. Recently, the ROAST consensus statement in 2019 established common approaches for LAS clinical assessment [19]. However, it has been agreed that most LAS (grades I–III) can be managed without surgery [20], with several different treatments recommended. Ultrasound imaging continues to play a crucial role in the assessment and management of this pathology [20].

Old conservative protocols, RICE (i.e., rest, ice, compression and elevation) and POLICE (i.e., protection, optimal loading, ice, compression and elevation) [21], were developed from existing data at the time that have now been partially refuted. Newer protocols such as PEACE (i.e., protection, elevation, avoid anti-inflammatory, compression and education) and LOVE (i.e., load, optimism, vascularization and exercise) [22] focus on education, vascularization, early loading and exercise. Therefore, new protocols have focused on active treatment such as exercise, while suggesting a de-emphasis on passive inflammatory control treatments.

Current conservative management strategies, identified in systematic reviews, include: avoiding non-steroidal anti-inflammatory drugs [22], electrical stimulation [23], ultrasound [24], ankle taping to improve joint position sense [25,26], balance training [27], early manual joint mobilization [28], static stretching [6] and exercise-based rehabilitation [29,30]. The target mechanism and impact of each of these interventions remain unclear but may include increasing dynamic neuromuscular control, pain reduction, improving postural sway and joint position sense and reducing the risk of re-injury [31,32,33,34].

Manual therapy has also been shown to be crucial in improving overall outcomes, with changes in ROM, bony alignment, pain reduction and overall physical function. A focus on talar positional faults has been a recent aim to improve ROM [35,36,37]. Modern neurophysiological theories suggest that the control of pain symptoms is useful for central and peripheral sensitization, which may help with LAS recovery [38]. In addition, the latest evidence has revealed the capability of manual therapy to avoid fibrosis after injury by reducing tissue levels of TGF-β1 [39].

Currently, evidence-based standards suggest a therapeutic approach for LAS that includes the use of therapeutic exercise. However, the specific treatment and dosage are still debated [27,28,40,41,42,43,44,45,46,47]. Manual therapy has been shown to be useful in LAS management [6,29,30,31,48,49,50]. A systematic review, including eight articles, found that, for acute ankle sprains, manual therapy diminished pain and increased dorsiflexion range of motion [48]; moreover, a recent systematic review with a meta-analysis [6], including seven trials, concluded that exercise-based rehabilitation reduces the risk of re-injury.

However, as previous systematic reviews and meta-analyses considered a broad research question regarding the efficacy of a certain treatment, and as they included studies that combined manual therapy with other interventions and compared it to different controls, it is difficult to assess how much additional benefit manual therapy adds to exercise therapy alone without providing precise evidence regarding whether manual therapy in addition to therapeutic exercise can lead to improved clinical outcomes. Indeed, to our best knowledge, no summary evidence studying the effect of manual therapy combined with therapeutic exercise exists. Our research hypothesis was that, as these two treatments have been shown to be effective when studied alone, the combination of manual therapy and therapeutic exercise could increase mostly clinical outcomes in patients with LAS.

The critical appraisal topic (CAT) [51,52] is a study design with a brief summary of the best evidence on a well-defined research question. It has the same methodology as the systematic review but is shorter (so it also includes fewer studies) and provides a detailed assessment of what is in the literature on that focused topic. The CAT has an important role to play in supporting evidence-based practice, in identifying gaps in knowledge and in quickly reviewing the literature and informing clinical professionals. Moreover, CAT can be used to answer specific, patient-orientated questions that arise recurrently in real-life practice [52]. However, the CAT provides only a qualitative summary of the results of the articles included. Therefore, performing a meta-analysis could also provide a quantitative estimate on the results of the included articles, helping clinicians to understand what the real effect of the studied intervention could be.

Therefore, this CAT with a meta-analysis aims to review the current literature on the efficacy of manual therapy plus therapeutic exercise, providing quantitative data through a meta-analysis.

## 2. Materials and Methods

### 2.1. Focused Clinical Question

Does manual therapy plus therapeutic exercise lead to pain reduction and functional improvement in subjects with an LAS, compared with therapeutic exercise alone?

### 2.2. Search Strategy

A systematic literature search was completed in MEDLINE (though the PubMed interface), PEDro, EMBASE and CINAHL from their inception to September 2021. The databases were interrogated with keywords and MeSH terms following the PICOT framework:

- **P**opulation: patients with an LAS;
- **I**ntervention: manual therapy plus therapeutic exercise;
- **C**omparison: therapeutic exercise;
- **O**utcome: clinical outcomes (i.e., pain, joint mobility and lower limb function);
- **T**ype of study: randomized clinical trial (RCT).

The search strings for each database are reported in Table 1. Moreover, cross-referencing of the included articles was performed in order to retrieve other articles.

### 2.3. Inclusion Criteria and Exclusion Criteria

Studies were included in this CAT if:

- Patients with an LAS were enrolled;
- The effect of manual therapy plus therapeutic exercise, compared with therapeutic exercise alone, was investigated;
- The study design was as an RCT;
- The publication languages were English or Italian.

Studies were excluded if:

- Patients with ankle instability or chronic ankle instability (CAI) were enrolled;
- The effects of only manual therapy or only therapeutic exercise were investigated;
- The study design was a cohort, non-randomized clinical trial or not identified as an RCT.

### 2.4. Evidence of Quality Assessment

The quality assessment was appraised using the Risk of Bias 2.0 (RoB), developed by the Cochrane Collaboration [53].

The Strength of Recommendation Taxonomy (SORT) [54] was used to assess the grade of evidence and to provide the strength of recommendation:

- A is assigned to consistent, high-quality patient-oriented evidence;
- B is assigned to evidence that is inconsistent or limited quality patient-oriented evidence;
- C is assigned to evidence that is considered an opinion, disease-oriented or a case series.

### 2.5. Data Synthesis

To assess the effect size (ES), i.e., the number that measures the strength of the relationship between two variables in a population or a sample estimate of that quantity, the mean difference (MD) was used when the same outcome measures were used across the included studies, and the standardized mean difference (SMD) with the Hedges “g” correction was used when different measurement instruments were used in the studies.

## 3. Results

### 3.1. Summary of Search and Key Findings

**Figure 1:**
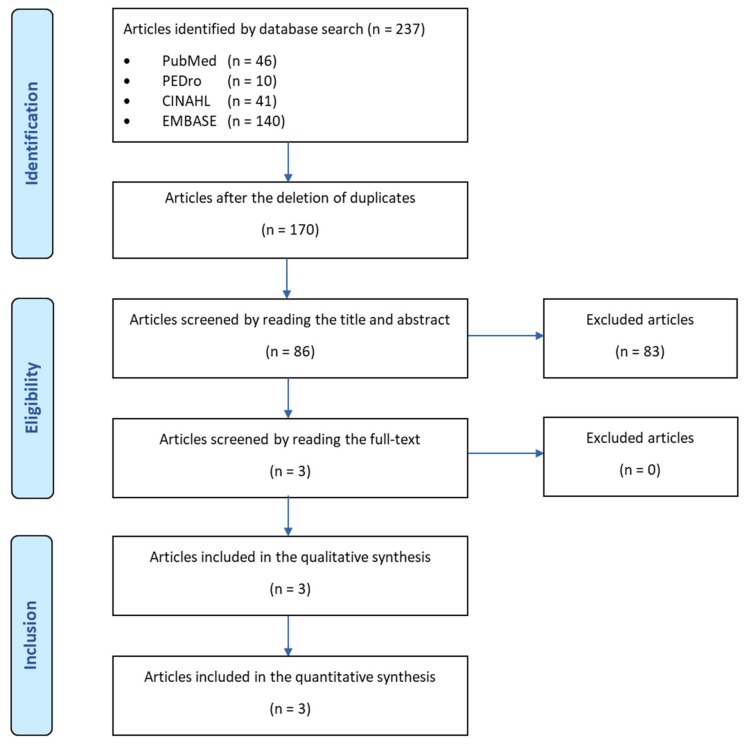
Flow chart of the research procedures.

Of 237 articles retrieved with the literature search, 3 articles were included in this study. A flow chart of the research procedure is reported in Figure 1.

A total of three studies were included and are briefly summarized below, and the detailed characteristics of the included studies are reported in Table 2.

Plaza-Manzano et al. [55] included an experimental group that performed proprioceptive and strengthening exercises consisting of four sessions with six exercises that were repeated twice a week. The exercises progressed every week and were supervised by two physiotherapists with at least 6 years of experience in Sports Physiotherapy. The manual therapy protocol consisted of mobilization applied with large-amplitude passive movements (grade III) repeated 10 times (the duration of techniques was 20–30 s with 2 min of resting), by two physiotherapists expert in manual therapy. The control group performed 4 weeks of proprioceptive and strengthening exercises. The addition of manual therapy to the proprioceptive and strengthening exercise elicited lower pain levels, reduced self-reported ankle instability, greater ankle strength, a lower pressure pain threshold (PPT) and a greater active range of motion (AROM) in patients with an LAS. The effect size of the visual analogue scale (VAS) and Cumberland Ankle Instability Tool (CAIT) was strong, with Cohen values of d = 1.23 and d = 1.45. PPT and ankle flexion and extension strength showed effect size values from moderate to strong (d = 0.65, d = 0.6, d = 0.65, d = 0.59, d = 0.9, d = 0.88). Within-group differences revealed that both groups exhibited significantly decreased PPTs at all of the pressure points and increased the ankle muscle strength compared to the baseline (*p* < 0.001). The mixed model linear analysis revealed a significant group by time interaction for the AROM in the ankle, which indicated that the experimental group exhibited higher ankle flexion (F = 21.93, *p* < 0.001) and extension values (F = 38.79, *p* < 0.001) with moderate effect sizes (d = 0.58 and d = 0.68).

In the work of Truyols-Dominiguez et al. [56], both groups received the same manual therapy protocol (four sessions, once a week, for 4 weeks), which included ankle and foot mobilization (non-thrust) applied at grades III–IV, delivered for 20–30 s with thrust manipulation, along with exercise. The experimental group also received myofascial therapy (pressure-release techniques, static strokes and cross-hand interventions) applied three times over the gastrocnemius and tibialis anterior muscles. The addition of myofascial techniques to a protocol of thrust/non-thrust manipulation and exercise resulted in statistically significant improvement in pain and function. The two by three mixed model analysis of variance revealed significant group by time interactions for pain (F = 11.727, *p* < 0.001) and functional score (F = 10.466, *p* < 0.002) with the treatment group that experienced a greater reduction in pain and improvement in function, observed 4 weeks after intervention (*p* < 0.001) and with a 1-month follow-up (*p* < 0.003). Between-group effect sizes were strong for both outcomes, with Cohen values of d > 1.3. Ankle flexion, ankle extension, ROM and PPT exhibited a great improvement in the experimental group, with effect size values of d > 0.85.

In the work of Cleland et al. [37], the MTEX group was treated by two physical therapists twice weekly for 4 weeks, and each session lasted 30 min. Manual therapy techniques consisted of thrust and non-thrust manipulation performed for 5 to 30 s bouts at grades I, II, III and IV. Patients were also instructed to perform two self-mobilization techniques at home and strengthening exercises. For 4 weeks daily, the HEP group performed mobilizing exercises for the foot and ankle, gentle stretching exercises, resistive band exercises, one-leg standing activities, standing on a balance board and weight-bearing functional activities. The addition of manual therapy to therapeutic exercise provided significant improvements in pain and function, both in the short- and in long-term follow-up. The overall group by time interaction for the mixed model ANOVA was statistically significant for Foot and Ankle Ability Measure (FAAM) ADL, FAAM sport, Lower Extremity Functional Scale (LEFS) and Numerical Pain Rating Scale (NPRS), exhibiting great outcomes of improvement in the MTEX group with the following values at 4 weeks: FAAM ADL = mean difference 11.7 with CI 95%: 7.4/16.11; FAAM sport = mean difference 13.3 with CI 95%: 8\18.6-LEFS = mean difference 12.8 con CI 95%: 9.1/16.5; and NPRS = mean difference −1.2 with CI 95%: −1.5/−0.90. The Mann–Whitney U test revealed a significant difference in favor of the MTEX group for the GRC (*p* < 0.001) at 4 weeks with the following values: MTEX group mean = 4.1; MTEX median = 4.0; MTEX mode = 4.0; HEP group mean = 3.0; HEP median = 3.0; and HEP mode = 3.0. The RCT synthesis showed that the combination of manual therapy and therapeutic exercise, compared to isolated exercise, was statistically significant, and the effect size was moderate to strong in the short-term function and pain domain. It is evident that myofascial therapy has a statistically significant contribution, but that does not exceed the minimal clinical important difference (MCID).

### 3.2. Results of Quality Assessment from the Best Available Evidence

The ROB 2 assigns a low risk of bias score for each domain, confirming the high quality of included studies. Moreover, the resulting force of the recommendation evidence was graded as 1A according to SORT (Table 3).

### 3.3. Results of the Meta-Analysis

**Figure 2:**
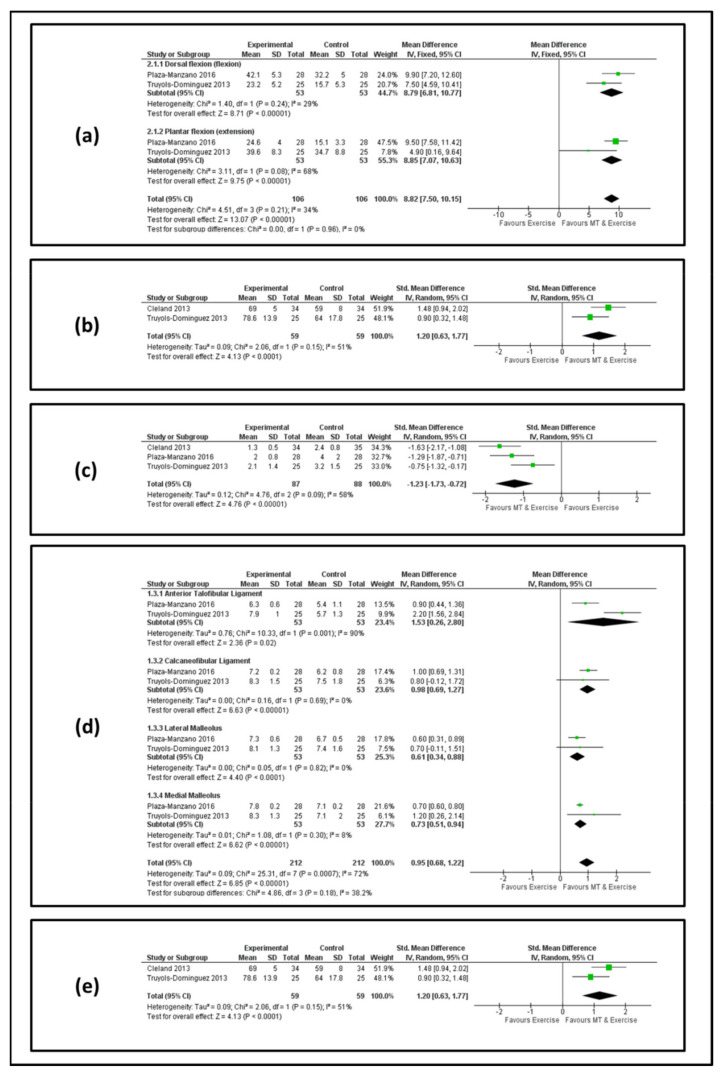
Forest plot of different outcomes between MT and MTEX at the end of the treatment. Forest plot demonstrating significant differences in items of active range of motion (**a**), lower limb function (**b**), pain (**c**), pain pressure threshold (**d**) and ankle stability (**e**).

**Figure 3:**
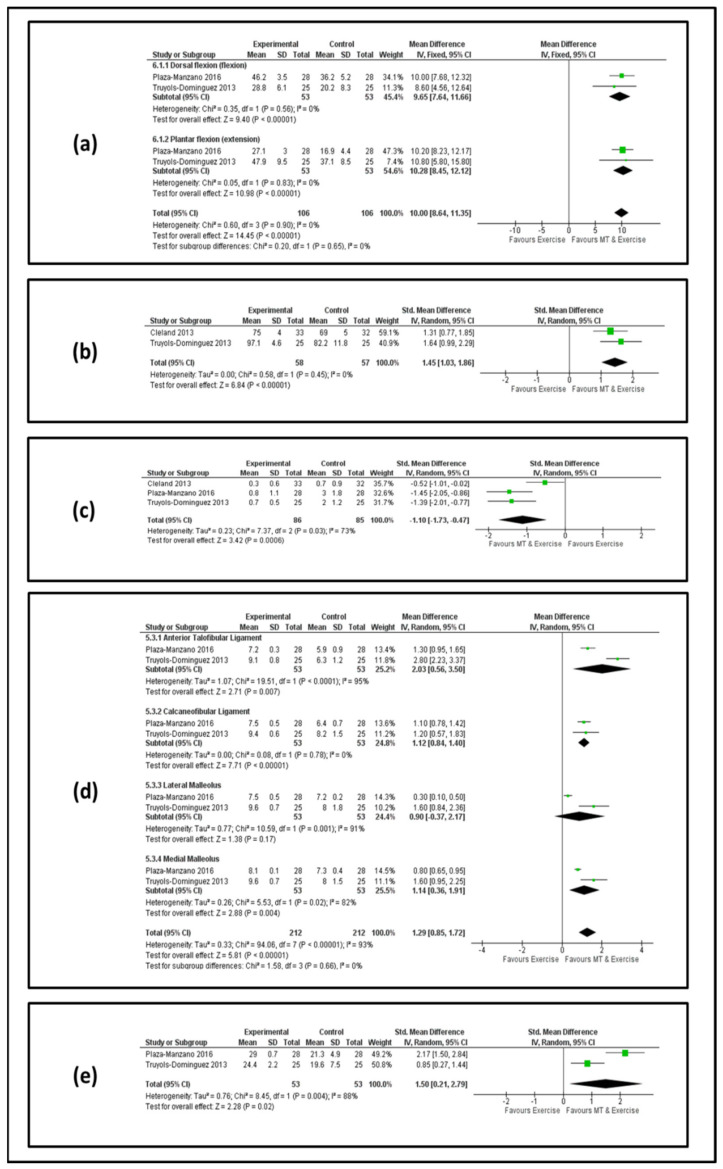
Forest plot of different outcomes between MT and MTEX at follow-up. Forest plot demonstrating significant differences in items of active range of motion (**a**), lower limb function (**b**), pain (**c**), pain pressure threshold (**d**) and ankle stability (**e**).

Figure 2 and Figure 3 show forest plots for AROM, lower limb function, pain, pain pressure threshold and ankle stability at the end of the treatment and at the follow-up, respectively.

*AROM.* Two studies [55,56] involving 106 patients reported effects on AROM for dorsal and plantar flexion. At the end of the treatment, we observed an MD value of 8.79 (95% confidence interval [IC] 6.81, 10.77) and 8.85 (95% CI 7.07, 10.63) in favor of manual therapy and exercise for dorsal and plantar flexion, respectively (Figure 2a). At the follow-up, the analysis showed an MD value of 9.65 (95% CI7.64, 11.66) and 10.28 (95% CI 8.45, 12.12) in favor of manual therapy and exercise for dorsal and plantar flexion, respectively (Figure 3a).

*Lower limb function.* Two studies [37,56] involving 118 patients reported effects on lower limb function. At the end of the treatment, we observed an MD value of 1.20 (95% IC 0.63, 1.77) in favor of manual therapy and exercise (Figure 2b). At the follow-up, the analysis showed an MD value of 1.45 (95% IC 1.03, 1.86) in favor of manual therapy and exercise (Figure 3b).

*PAIN.* Three studies [37,55,56] involving 175 patients reported effects on pain. At the end of the treatment, we observed an MD value of −1.23 (95% IC −1.73, −0.72) in favor of manual therapy and exercise (Figure 2c). At the follow-up, the analysis showed an MD value of −1.10 (95% IC −1.73, −0.47) in favor of manual therapy and exercise (Figure 3c).

*PPT.* Two studies [55,56] containing 106 patients were eligible for the data analysis of the anterior talofibular ligament, calcaneofibular ligament, lateral malleolus and medial malleolus. Regarding the anterior talofibular ligament, at the end of the treatment, we observed an MD value of 1.53 (95% IC 0.26, 2.80) in favor of manual therapy and exercise (Figure 2d), and at the follow-up, the analysis showed an MD value of 2.03 (95% IC 0.56, 3.50) in favor of manual therapy and exercise (Figure 3d). Regarding the calcaneofibular ligament at the end of the treatment, we observed an MD value of 0.98 (95% IC 0.69, 1.27) in favor of manual therapy and exercise (Figure 2d), and at the follow-up, the analysis showed an MD value of 1.12 (95% IC 0.84, 1.40) in favor of manual therapy and exercise (Figure 3d). Regarding the lateral malleolus at the end of the treatment, we observed an MD value of 0.61 (95% IC 0.34, 0.38) in favor of manual therapy and exercise (Figure 2d), and at the follow-up, the analysis showed an MD value of 0.90 (95% IC −0.37, 2.17) in favor of manual therapy and exercise but without statistical significance (Figure 3d). Regarding the medial malleolus at the end of the treatment, we observed an MD value of 0.73 (95% IC 0.51, 0.94) in favor of manual therapy and exercise (Figure 2d), and at the follow-up, the analysis showed an MD value of 1.14 (95% IC 0.36, 1.91) in favor of manual therapy and exercise (Figure 3d).

*Ankle Stability.* Data from two studies [37,56] involving 118 patients were selected for the meta-analysis. At the end of the treatment, we observed an MD value of 1.20 (95% IC 0.63, 1.77) in favor of manual therapy and exercise (Figure 2e). At the follow-up, the analysis showed an MD value of 1.50 (95% IC 0.21–2.79) in favor of manual therapy and exercise (Figure 3e).

### 3.4. Clinical Bottom Line

The CAT evidence suggests that incorporating manual therapy into therapeutic exercise improves short- and long-term function and decreases short- and long-term pain, assessed with the outcome measures of FAAM, VAS, AROM, NPRS, PPT, CAIT and LEFT, compared to therapeutic exercise alone.

The comprehensive analysis of data is described in detail in Table 2 and Table 3.

## 4. Discussion

The objective of this CAT was to critically assess and summarize the evidence on the effect of manual therapy and therapeutic exercise compared with therapeutic exercise alone. This work showed that manual therapy with therapeutic exercise improves dorsiflexion and plantar flexion, lower limb function and pain when compared with therapeutic exercise alone.

It is necessary to specify that the term “manual therapy” and “therapeutic exercise” may be defined as “umbrella terms”, including a multitude of therapeutic interventions that could be included. In the articles reviewed, manual therapy included mobilizations of articular and nerve structures, manipulations (thrust and non-thrust) and myofascial therapy (pressure-release techniques, static and dynamic strokes and cross-hand intervention). Therapeutic exercise included home and clinic-based exercise focused on range-of-motion improvement, strength, balance, proprioception recovery and pain reduction. In the work of Plaza-Manzano et al. [55], the manual therapy protocol consisted of mobilization (talocrural distraction, talocrural anterior-posterior (AP) glide, distal tibiofibular AP glide and superficial peroneal nerve mobilization), applied to grade III, which included large-amplitude passive movements repeated 10 times (technique duration was 20–30 s with 2 min of rest), performed by two therapists who were experts in manual therapy.

In this CAT, RCTs including patients with an acute LAS associated with inflammatory symptoms such as swelling and pain were included. We also specify that which is shown in Table 2. Patients recruited by Cleland et al. [37] and Truyols-Dominiguez et al. [56] presented a first episode of LAS. Plaza-Manzano et al. [55] recruited patients with a recurrence of LAS, but they always considered the acute event and excluded the presence of any type of functional instability.

The study by Plaza-Manzano et al. [55] reported that the addition of manual therapy to the proprioceptive and strengthening exercises elicited better results. However, it was noted that the isolated exercise program was also associated with improvements across all of the variables examined in this study. This finding suggests that proprioceptive and strengthening exercises are useful in the management of the ankle sprains, but the inclusion of manual therapy may maximize treatment efficacy. This result agrees with that of Schiftan et al. [57], who suggested that proprioception and strengthening are key parameters in ankle sprain rehabilitation in terms of the prevention of recurrent injuries, particularly in the sporting population [58,59].

Truyols-Dominiguez et al. [56] included mobilizations (talo crural AP glide, hindfoot lateral glide and distal tibiofibular AP glide) applied to grades III-IV for 20–30 s, manipulations (talo crural distraction thrust technique and proximal tibiofibular thrust technique) and myofascial therapy only for the experimental group, performed three times (pressure release techniques of the gastrocnemius muscle, static stroke of the fibularis and tibial muscle and cross-hand techniques of gastrocnemius muscle). In the work of Cleland et al. [37], manual therapy techniques consisted of manipulations (talo crural distraction thrust, talo crural AP glide, weight bearing talo crural AP mobilization with movement, hindfoot lateral glide, proximal tibiofibular thrust technique and distal tibiofibular AP glide) executed for 30 s at grades I, II, III and IV. Even therapeutic exercise included a multitude of interventions, such as: foot and ankle self-mobilizations, stretching, strengthening exercises with elastic bands or weights (isometric, isotonic and plyometric), motor control exercises and functional exercises.

The results by Cleland et al. [37] suggest that a manual therapy and exercise (MTEX) approach is superior to a home exercise program (HEP) in the treatment of ankle sprains, which is in accordance with the study conducted by Truyols-Dominiguez et al. [56] and Plaza-Manzano et al. [55]. The study by Truyols-Dominiguez et al. provides evidence that, in the treatment of individuals post-inversion ankle sprain, the addition of myofascial therapy to a plan of care consisting of thrust and non-thrust manipulation and exercise may further improve outcomes compared to a plan of care solely consisting of thrust and non-thrust manipulation and exercise.

Moreover, the study conducted by Plaza-Manzano et al. suggests that the addition of manual mobilization on the ankle, which may also influence the dorsolateral peripheral nerves of the foot, to the proprioceptive and strengthening exercises elicited lower pain levels, reduced self-reported functional ankle instability, greater ankle strength, lower pressure pain thresholds and greater active ranges of motion in patients with recurrent ankle sprains compared to proprioceptive and strengthening exercises alone.

This CAT highlights the effectiveness of manual therapy with therapeutic exercise. This result met our research hypothesis. Manual therapy has been used to restore normal ROM, reduce local ischemia, stimulate proprioception, break fibrous adhesions and reduce pain. Several studies have assessed the efficacy of various manual therapy treatments in patients with lateral ankle sprains. Eisenhart et al. [60] showed a one-time manipulation in an acutely sprained ankle to the talocrural joint resulted in improved ROM over a control group but no significant reduction in pain. Another study showed that mobilization to the talocrural joint resulted in improved AROM and earlier return to work in patients with subacute to chronic lateral ankle sprains [61]. Moreover, the joint mobilization techniques showed improvements in dorsiflexion range. Collins et al. [62] reported improvements in joint mobility in patients with subacute ankle sprains, after the further sliding of the talus was combined with active movement and weight bearing. Vicenzino et al. [36] identified improvements after the completion of 10 oscillations with the same technique in a small population with chronic ankle sprains. Yeo and Wright [63] identified improved ranges of motion after three sessions in subjects with ankle sprains during the subacute phase, using higher a slip talus without a weight bearing. Finally, Beazell et al. [64] identified the similar effects after performing sessions of four mobilizations compared with manipulation in 43 patients with chronic ankle sprains. Taking into account therapeutic exercise, several studies have shown effectiveness in different ankle pathologies. Davenpor et al. [65] studied the clinical effects of hands-on passive stretching treatment procedures directed to the talocrural joint that vary in treatment speed during the post-acute injury period, compared to hands-on placebo control intervention. Moreover, proprioceptive training programs were effective in reducing the incidence rates of ankle sprains in the athletic population, including those with and those without a history of ankle sprains [66]. Therefore, according to our results and to previous researchers, clinicians can use manual therapy in addition to therapeutic exercise to improve pain, lower limb function and range of motion.

Hence, we suggest that all the three selected research studies were in accordance with each other and suggest that combining therapeutic exercises with manual therapy help in reductions in pain, increase ankle strength and stability as well as improve the function of recurrent ankle sprains both at the end of treatment and after a follow-up.

The studies included in this CAT administered proprioceptive training. Some recent studies have highlighted the importance of proprioceptive rehabilitation in the monopodalic stance with the use of unstable electronic platforms for the prevention of ankle injuries in professional basketball players and for reductions in the number of injuries, training sessions and lost games [67]. The improvement of proprioceptive neuromuscular control and the great exposure of capsules and ligaments to repeated tensile forces increase their capacity to absorb these, perhaps when neuromuscular control is insufficient, further reducing the risk of injury [67,68]. With increases in age, this type of therapeutic proposal is strongly influenced by sight, suggesting that it works with closed eyes [69].

Our findings should be interpreted carefully according to the following limitations of the included studies: (1) the inadequacy of only the active sports population for generalization [55]; (2) the lack of a pure control group or placebo [37] and (3) the presence of attentional bias [56]. Finally, the search on only four databases could be a limitation of this research; however, an article [70] showed that most RCTs in physiotherapy were indexed by PEDro, EMBASE, PubMed and CINAHL. Therefore, this search may have been able to find all potential articles on this topic.

Other unexplored aspects of included studies are psychosocial factors that may play a key role in achieving better outcomes in a shorter time or that may adversely affect recovery, leading to chronic pain condition. It is recommended that future studies consider larger sample sizes, with a longer follow-up and with the inclusion of psychosocial outcomes.

In clinical practice, health professionals should consider the inclusion of manual therapy combined with therapeutic exercise in LAS rehabilitation programs to improve outcomes. The exact mechanisms by which these outcomes are achieved remain an open area of investigation.

The purpose of this CAT is to advance our understanding of how manual therapy may contribute to improved outcomes when combined with therapeutic exercise for people with an LAS. These research findings may help to refine future clinical practice guidelines.

In conclusion, considering the strong evidence regarding the best choice for the management of lateral ankle sprains based on progressive load and therapeutic exercise, in this CAT, we investigated whether the association of manual therapy techniques with therapeutic exercise could improve outcomes in the short and medium term. Our results demonstrate that the association of manual therapy with therapeutic exercise is able to improve clinical outcomes compared to therapeutic exercise alone. Therefore, clinicians should use both treatments as recommended by recent clinical practice guidelines on LASs [71] to reduce swelling and pain, improve foot and ankle mobility and normalize walking parameters in patients with an LAS.

## Figures and Tables

**Table 1:** Search strings for each database.

Database	Search String
MEDLINE	(“Ankle Injuries”[Mesh] or (ankle injur*) OR (ankle sprain*) or (ankle strain*)) AND (“Exercise Therapy”[Mesh] OR “Exercise”[Mesh] or exercis*) AND (“Musculoskeletal Manipulations”[Mesh] or (musculoskeletal manipulation*) or (manual therap*) or (manipulative therap*))
PEDro	ankle sprain* manual therap* exercis*
EMBASE	(‘ankle injuries’/exp OR (ankle AND injur*) OR ‘ankle sprain’/exp OR (ankle AND sprain*) OR (ankle strain*)) AND (‘exercise’/exp OR exercis*) AND (‘manual therapy’/exp OR (manual AND therap*))
CINAHL	(ankle AND (sprain* OR injur*)) AND (exercis*) AND (manual therap*)

**Table 2:** Detailed characteristics of the included studies.

	Plaza-Manzano et al. 2016 [55]	Truyols-Dominiguez et al. 2013 [56]	Cleland-Minkten et al. 2013 [37]
Study design	Randomized controlled trial	Randomized controlled trial	Randomized controlled trial
Partecipants	N = 56 Age = 24 ± 2.5 Gender = 39 M–17 F Patients were randomly recruited from the University Hospital of Madrid	N = 50 Age = 33 ± 10 Gender = 37 M–13 F Patients were randomly recruited from a physical therapy clinic in Madrid	N = 74 Age = 35.1 ± 11 Gender = 38 M–36 F Patients were randomly recruited from 4 physical therapy clinics in the USA
Inclusion criteria	- previous initial PFI ankle sprain graded I, II, III, at least 12 months prior to the study beginning, associated with inflammatory symptoms such as swelling and pain - recurrence of previous PFI ankle sprains - patients had not sprained the affected ankle in the last 3 months - regular sport practice	- patients had to be between 18 and 50 years old - patients with their first inversion ankle sprain grade I or II, injured for less than 5 days	Patients with an inversion ankle sprain grade I or II, with an NPRS score greater than 3 and a negative result from the Ottawa ankle rules
Exclusion criteria	- surgical treatments - previous fractures in either lower extremity - adjacent pathologies that disturbed joint integrity or function And required at least one interrupted day of desired physical activity	- previous trauma - fracture - surgery to lower extremity - any concomitant lower extremity pathology (vascular disease, osteoarthritis) - pregnancy - painful medical syndrome (fibromyalgia, rheumatoid arthritis, whiplash, carpal tunnel syndrome) - the use of medication within 7 days prior the study - previous physical therapy intervention provided for the foot region	- contraindication to manual therapy (tumor, fracture, rheumatoid arthritis, osteoporosis, prolonged history of steroid use, severe vascular disease) - surgery of the distal fibula, ankle joint or rearfoot - insufficient English language skills to complete all questionnaires - inability to comply with the treatment and follow-up schedule
Intervention investigated	The experimental group performed 4 weeks of exercises combined with manual therapy (mobilization to influence joint and nerve structures). The control group performed 4 weeks of proprioceptive and strengthening exercises. The proprioceptive and strengthening exercise protocol consisted of four sessions of six exercises that were repeated twice a week and progressed every week, supervised by two physiotherapists with at least 6 years of experience in Sports Physiotherapy; The manual therapy protocol consisted of mobilization applied at grade III, including large amplitude passive movements repeated 10 times (the duration of techniques was 20–30 s with 2 min of resting) by two physiotherapists who were experts in manual therapy.	The experimental group received the thrust and non-thrust manipulation and exercise protocol in addition with myofascial manual therapy techniques for four sessions, once per week, for 4 weeks. The control group received the same thrust and non-thrust manipulation and exercise protocol for four sessions, once per week, for 4 weeks. Both groups received the same manual therapy protocol (performed for four sessions, once per week, for 4 weeks), which included ankle and foot mobilization (non thrust) applied at grade 3–4 and delivered for 20–30 s for thrust manipulation and exercise. Only the experimental group received myofascial therapy (pressure-release techniques, static strokes and cross-hand interventions) applied three times over the gastrocnemius and tibialis anterior muscles.	Patients in the MTEX group (manual therapy and exercise) were treated twice a week for 4 weeks, and each treatment session included thrust and non-thrust manipulation and mobilizing and strengthening exercises. Patients in the HEP group (home exercise program) attended physical therapy for four sessions for the instruction and progression of strengthening and proprioceptive exercises. The MTEX group was treated by two physical therapists twice weekly for 4 weeks, and each session lasted 30 min. Manual therapy techniques consisted of thrust and non-thrust manipulation performed for 5–30 s bouts at grades I, II, III and IV. Patients were also instructed to perform two self-mobilization techniques at home and strengthening exercises. For 4 weeks daily, the HEP group performed mobilizing exercises for the foot and ankle, gentle stretching exercises, resistive band exercises, one-leg standing activities, standing on a balance board and weight-bearing functional activities.
Outcome measure	Assessors measured the outcomes (VAS, CAIT, PPT, Active range of motion measured with goniometer, Strength in ankle flexion and extension with dynamic dynamometry) before and after the 4 weeks of treatment.	Assessors measured the outcomes (NPRS, Functional score for the assessment of acute lateral ankle sprains, Active range of motion measured with goniometer, PPT) at the baseline after the last treatment session and at 1 month follow-up.	Assessors measured the outcomes (FAAM, LEFS, NPRS, GRC scale) at the final physical therapy session (4 weeks) and after 6 months.
Results	The addition of manual therapy to the proprioceptive and strengthening exercise elicited lower pain levels, reduced self-reported ankle instability, greater ankle strength, lower PPT and greater AROM in patients with an LAS. The effect size was large to moderate in the following domains: - CAIT e VAS large effect size - PPT moderate effect size - AROM moderate effect size	The addition of myofascial techniques to a protocol of thrust/non-thrust manipulation and exercise resulted in statistically significant improvements in pain and function. The effect size between groups was large to moderate in all domains (pain, instability, weight bearing, swelling, walking pattern).	The addition of manual therapy to therapeutic exercise resulted in significant improvements in pain and function in both the short- and long-term follow-up. The effect size between groups was large to moderate (NPRS, FAAM, LEFS, GRC).
Level of evidence	1b	1b	1b
Quality assessment score	ROB-2: Low risk of Bias	ROB-2: Low risk of Bias	ROB-2: Low risk of Bias
Contribution to CAT question	Conclusive contribution	Conclusive contribution	Conclusive contribution

**Table 3:** Risk of bias assessment of the included studies.

Study	Bias Arising from the Randomization Process	Bias Due to Deviations from Intended Interventions	Bias Due to Missing Outcome Data	Bias in the Measurement of the Outcome	Bias in the Selection of the Reported Result	Overall Bias
Plaza-Manzano et al., 2016 [55]	LOW	LOW	LOW	LOW	LOW	LOW
Truyols-Dominiguez et al., 2013 [56]	LOW	LOW	LOW	LOW	LOW	LOW
Cleland et al., 2013 [37]	LOW	LOW	LOW	LOW	LOW	LOW

## Data Availability

Data generated or analyzed during this study are included in this published study. Other information of this study is available from the corresponding author on reasonable request.
